# Hypoglycemia in Infants and Effect on Neurodevelopment

**DOI:** 10.15844/pedneurbriefs-34-18

**Published:** 2020-12-18

**Authors:** Bridget R. McGowan, Tracy S. Gertler

**Affiliations:** 1 Division of Neurology, Ann & Robert H. Lurie Children's Hospital of Chicago, Chicago, IL; 2 Departments of Pediatrics and Neurology, Northwestern University Feinberg School of Medicine, Chicago, IL

**Keywords:** Hypoglycemia, Child Development, Blood Glucose, Infant, Newborn

## Abstract

In a prospective, randomized treatment trial, investigators from multiple institutions in the HypoEXIT Study Group investigated the developmental outcomes after neonatal hypoglycemia, comparing the traditional glucose threshold 47 mg/dL vs. 36 mg/dL.

In a prospective, randomized treatment trial, investigators from multiple institutions in the HypoEXIT Study Group investigated the developmental outcomes after neonatal hypoglycemia, comparing the traditional glucose threshold 47 mg/dL vs. 36 mg/dL. Healthy infants without initial severe hypoglycemia (<35 mg/dL) but with asymptomatic moderate hypoglycemia between 3-24 hours of life were randomly assigned to the lower threshold group or, the higher threshold group. The study demonstrated non-inferiority in the lower threshold vs. traditional threshold group regarding Bayley III scores at 18 months. [1]

COMMENTARY. Management of hypoglycemia in the newborn period is highly variable among institutions and professional societies. 2011 AAP guidelines define neonatal hypoglycemia as blood glucose <47 mg/dL and recommend maintaining blood glucose >40 mg/dL in the first 4 hours and >45 between hours 4-24 [2]. The Pediatric Endocrine Society has an even stricter threshold of >50 mg/dL [3]. However, a higher cutoff necessitates additional interventions, as seen in this study. Both prolonged hyper and hypoglycemia have been associated with poor neurologic outcomes. However, less research has been done into the impact of more transient hypoglycemia episodes, which are generally thought to be insignificant [4]. As this paper demonstrates no difference in Bayley scoring at 18 months, perhaps added interventions to match a higher threshold are not needed. This study's strength is that it is prospective and randomized, rather than observational like many previous, similar studies. Also, as multiple centers enrolled and were instructed to tailor supplementation to their usual practices, this study focuses on the main effect and has greater applicability. The literature includes multiple contradictory studies suggesting that even moderate hypoglycemia impacts executive function [5] and cognitive functioning [6], evidenced at later ages by poor school performance [7]. A major limitation of this study is the single test and age used to evaluate cognitive function. Early assessment precludes testing of speech, comprehension, and motor planning previously seen to be impacted in other studies. Second, there have been varying definitions of “neurologic impairment” ranging from specific testing like the Bayley (as in this paper) to ICD diagnostic codes ranging from autism spectrum disorder to febrile seizures [6], suggesting a lack of insight into the brain microcircuitry most at risk. Third, the authors recognize the potential for recurrent hypoglycemia, which may be more severe if starting at a lower threshold. Data are lacking from the biochemical literature on whether the brain is most susceptible to injury from repeated drops in glucose stores or total time spent in a hypoglycemic state.

Taken together, this paper reiterates the need for a critical reevaluation of treatment pathways based on lab values alone and the need to tailor treatment based on the clinical picture.

## Disclosures

The authors have declared that no competing interests exist.
